# Sequencing analysis of 20,000 full-length cDNA clones from cassava reveals lineage specific expansions in gene families related to stress response

**DOI:** 10.1186/1471-2229-7-66

**Published:** 2007-12-20

**Authors:** Tetsuya Sakurai, Germán Plata, Fausto Rodríguez-Zapata, Motoaki Seki, Andrés Salcedo, Atsushi Toyoda, Atsushi Ishiwata, Joe Tohme, Yoshiyuki Sakaki, Kazuo Shinozaki, Manabu Ishitani

**Affiliations:** 1 Metabolomics Research Group, RIKEN Plant Science Center, 1-7-22 Suehiro-cho, Tsurumi-ku, Yokohama, 230-0045, Japan; 2 Agrobiodiversity and Biotechnology Project, International Center for Tropical Agriculture (CIAT), A.A. 6713, Cali, Colombia; 3 Plant Functional Genomics Research Group, RIKEN Plant Science Center, 1-7-22 Suehiro-cho, Tsurumi-ku, Yokohama, 230-0045, Japan; 4 Genome Core Technology Facilities, RIKEN Genomic Sciences Center, 1-7-22 Suehiro-cho, Tsurumi-ku, Yokohama, 230-0045, Japan

## Abstract

**Background:**

Cassava, an allotetraploid known for its remarkable tolerance to abiotic stresses is an important source of energy for humans and animals and a raw material for many industrial processes. A full-length cDNA library of cassava plants under normal, heat, drought, aluminum and post harvest physiological deterioration conditions was built; 19968 clones were sequence-characterized using expressed sequence tags (ESTs).

**Results:**

The ESTs were assembled into 6355 contigs and 9026 singletons that were further grouped into 10577 scaffolds; we found 4621 new cassava sequences and 1521 sequences with no significant similarity to plant protein databases. Transcripts of 7796 distinct genes were captured and we were able to assign a functional classification to 78% of them while finding more than half of the enzymes annotated in metabolic pathways in Arabidopsis. The annotation of sequences that were not paired to transcripts of other species included many stress-related functional categories showing that our library is enriched with stress-induced genes. Finally, we detected 230 putative gene duplications that include key enzymes in reactive oxygen species signaling pathways and could play a role in cassava stress response features.

**Conclusion:**

The cassava full-length cDNA library here presented contains transcripts of genes involved in stress response as well as genes important for different areas of cassava research. This library will be an important resource for gene discovery, characterization and cloning; in the near future it will aid the annotation of the cassava genome.

## Background

Among starch producing crops, cassava (*Manihot esculenta *Crantz, Euphorbiaceae) has a higher carbohydrate production than rice or maize under suboptimal conditions [1]; more than 163 million tons are produced in the world each year and about 84% of them are used for direct human consumption and animal feed [2]. Cassava starch is used as a raw material for a wide range of food products and industrial goods, including paper, cardboard, textile, plywood, glue and alcohol [3]. Moreover, because starch production from cassava is cheap compared to other crops, it is gaining attention as a biomass source for fuel production [4]. The growing interest in cassava as an energy crop is evidenced by a genome sequencing project [5] and the increasing production and technical advancements in tropical countries; for instance, cassava fresh root production in Thailand increased from 6.3 to 20 million tons between 1973 and 1990 [6] while a 2.2% increase per year has been reported for the same period worldwide [2].

By virtue of its remarkable tolerance to abiotic stresses, cassava is grown in marginal, low fertility acidic soils showing increased nutrient use efficiency [7]. It is known to maintain a healthy appearance in drought-prone areas, remaining photosynthetically active though at a reduced rate [8]. Because cassava is very drought-resistant and the tubers can be left in the soil for a couple of years, it is considered an important reserve carbohydrate source to prevent or relieve famine [9]. Cassava has some unusual characteristics that make it highly productive in near optimum environments (hot-humid climates with high solar radiation), these include elevated activities of the C_4 _phosphoenolpyruvate carboxylase enzyme, long leaf life and low photorespiration rates [10]; it, however, is usually grown in marginal highly eroded soils with uncertain rainfall and almost no agrochemical input. Although cassava has some features that allow it to cope with stress better than other crops, e.g. high stomatal sensitivity to environmental humidity [11], deep rooting capacities and quick recovery after stress [12], under these conditions productivity is sub-optimal and unstable [10]. Cassava productivity is also threatened by bacterial and viral diseases [13], as well as arthropod pests [14]. Moreover, its high starch content is in contrast with its deficiency in proteins and key micronutrients (zinc, iron and vitamins), as well as the production of toxic hydrogen cyanide [15].

To address these issues, traditional breeding methods have had some success, particularly in improving fresh root yield and dry matter content under non-stress conditions [16], however, because of the crop's heterozygous genetic makeup and long growth cycle, progress with this approach is slow [17]. The use of biotechnology to improve cassava cultivars is a more straightforward strategy that relies on the tools of molecular and cell biology to find genetic determinants of desirable phenotypes [18]. The construction of genetic maps and the identification of quantitative trait loci have yielded some results in cassava response to biotic stress [19], yet, the identification of candidate genes with this approach is a time consuming process involving the construction of bacterial artificial chromosome (BAC) libraries and anchoring of these clones to the genetic map [20]. A reverse-genetics approach [21] can be a more direct solution, relying on the accumulated knowledge of gene function in model species it is possible to assess the effects of selected genes through regulation of their expression. As an example, silencing of P-450 cytochromes has allowed the production of cyanogen-free transgenic cassava plants [22,23].

One tool that may assist both, the characterization of a plant expressed genes and the isolation of nucleotide sequences of genes with known function, are ESTs [24]. These are a cost-effective gene discovery methodology that is also useful for the study of gene expression [25]. Despite its importance, large-scale sequence collections from cassava are scarce, there are 36162 expressed cassava sequences in the dbEST database [26] as of April 2007, which is a small number compared to the number of ESTs of maize (2961956), rice (1912256), soybean (686687), potato (275813) or sugarcane (257998). This is likely to change with the release of a cassava draft genome sequence this year by the United States Department of Energy's Joint Genome Institute. Although ESTs can aid the annotation of the cassava genome, the fact that most of them come from libraries made of random mRNA fragments, make them insufficient to accurately and fully define gene models [27], ESTs are not only derived from partial transcripts, but also they can confound alternatively spliced forms during the assembly process [28]. Moreover due to the fragmentary nature of ESTs, their use in gene functional analysis is limited [29-31].

Full-length cDNA libraries, on the other hand, are built in such a way that one insert represents one transcription unit, providing information on complete molecules for the functional dissection of genes [28]. We built a full-length enriched cDNA library from cassava leaves and roots subject to drought, heat, and acidic conditions, as well as from roots subject to post-harvest physiological deterioration (PPD), a major obstacle for cassava commercialization [32]. The aim of this library is to support research in cassava improvement for high yield under abiotic stress, providing full sequences of stress-responsive genes and expanding the gene catalog of this species. The characterization of the transcripts captured in the library and the selection of non-redundant clones will certainly aid the annotation of genomic sequences [30] and the construction of microarrays or other tools for functional genomics [33]. In order to characterize the library and find the number and putative functions of the transcripts captured, nearly 20.000 clones were sequenced from both ends, these ESTs, although unlikely to include the whole sequence of the inserts, are tagged with clone names. Because this information is considered during the assembly process, ESTs derived from a full-length library allow, in principle, a more accurate definition of transcript units than normal ESTs.

The annotation of the sequences acquired and the availability of the genome sequences of two species closely related to cassava such as castor bean (*Ricinus communis*, Euphorbiaceae) and poplar (*Populus trichocarpa*, Salicaceae) [34] as well as the complete set of genes from *Arabidopsis thaliana*, provide altogether an opportunity to study the evolution of the cassava genome by means of comparative genomics [35]; if it is possible to define gene correspondences between these species and on that basis find sequences that are unique to cassava, a closer inspection of these genes could provide hints as to the mechanisms underlying cassava's unique features. Cassava is believed to be an allotetraploid that appeared by hybridization of wild *Manihot *species [36], it would then be interesting to see what genes within a highly heterozygous gene pool have remained functional during cassava domestication; for this we use a methodology for the detection of recent duplications that is based on the detection of groups of genes sharing similarity to single sequences in other genomes, hopefully the genes detected with this strategy will aid cassava research for the genetic improvement of an already outstanding crop.

## Results

### Sequencing and assembly of both-end, single-pass sequences

A full-length cDNA library was constructed from leaves and roots of cassava plants under various environmental conditions (see methods), 19968 clones (CAS01_001_A01 to CAS01_052_P24 or 52 × 384-well plates) were sequenced from both ends; the clones are available at RIKEN Bioresource Center [37] and the sequences can be obtained from the DNA Databank of Japan (DDBJ) under accession numbers DB920056-DB955455.

Sequence reads were trimmed for low quality and vector contamination; 35400 sequences belonging to 19449 clones were obtained after this process. For the clones with 5' and 3' sequence data showing significant sequence similarity to known proteins, the calculated full-length ratio was 0.84, meaning that roughly 85% of the clones contain the complete coding sequence (CDS) of their inserts.

The sequences were assembled into 6355 contigs and 9026 singletons using CAP3; however, given that all sequences were tagged with their respective clone ids, we were able to further cluster the results of the CAP3 assembly to build 10577 scaffolds representing distinct transcripts. Of these, 2005 (19%) contained, in a single contig, both ends of the respective clones and were thus considered full-length sequences.

Alternative splicing variants had to be detected in order to estimate the number of different genes in the library; using the approach described in the methods section, we identified 4877 transcripts of just 2096 genes. We determined that the full-length library includes transcripts of a total of 7796 distinct genes, with alternative transcripts of about 26% of them. To find the number of new transcripts captured in this library, relative to the number of expressed sequences from cassava already present in GenBank, we conducted a BLASTN search of the sequences in our assembly against the 36162 EST sequences in dbEST as of April 2007. Any sequence with no hit to the database or with an e-value > 1e-100 and a percent identity < 95 % was considered to be a new cassava transcript. In this way we found 4621 new cassava sequences in our set. Furthermore, by running BLASTX against a UniProt – TrEMBL database of plant proteins, we found 1521 transcripts with no similarity (e-value 1e-5) to known proteins in other plant species (Table 1).

**Table 1:** Summary of library properties and assembly results after sequencing the clones from both ends.

**Library feature**	**Sequence count**
Clones	19968
Sequence reads (trimmed)	35400
Contigs	6355
Singletons	9026
Scaffolds	10577
Fully sequenced transcripts	2005
Distinct genes	7796
Novel cassava transcripts	6967
Novel plant transcripts	1521

**Figure 1:**
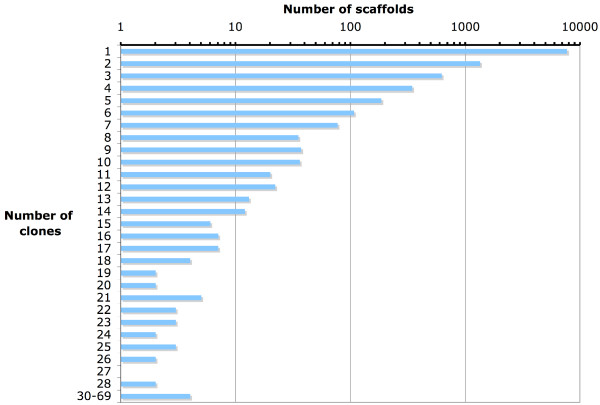
Cluster profile of the assembly of cassava ESTs. The graph presents the number of clones per assembled scaffold; it should be noticed that over 7000 transcripts are represented by a single clone in the full-length library.

The information in the CAP3 assembly and the names of the sequenced clones were used to build a cluster profile representing the number of clones per assembled scaffold (Figure 1); this was done in order to provide an approximation of the total number of cassava transcripts using the Compound Poisson process model implemented in the ESTstat package [38,39]. We obtained a number of 50698 transcripts, which is in the range of the number of transcripts estimated in poplar, Arabidopsis and rice (Table 2).

**Table 2:** Number of predicted transcripts according to the species-specific datasets downloaded from the given locations.

**Species**	**Predicted transcripts**	**Source**
*M. esculenta*	50698	This paper
*P. trichocarpa*	58036	Joint Genome Institute [105]
*A. thaliana*	31527	TAIR [106]
*O. sativa*	62827	TIGR [107]

### Sequence functional annotation

**Figure 2:**
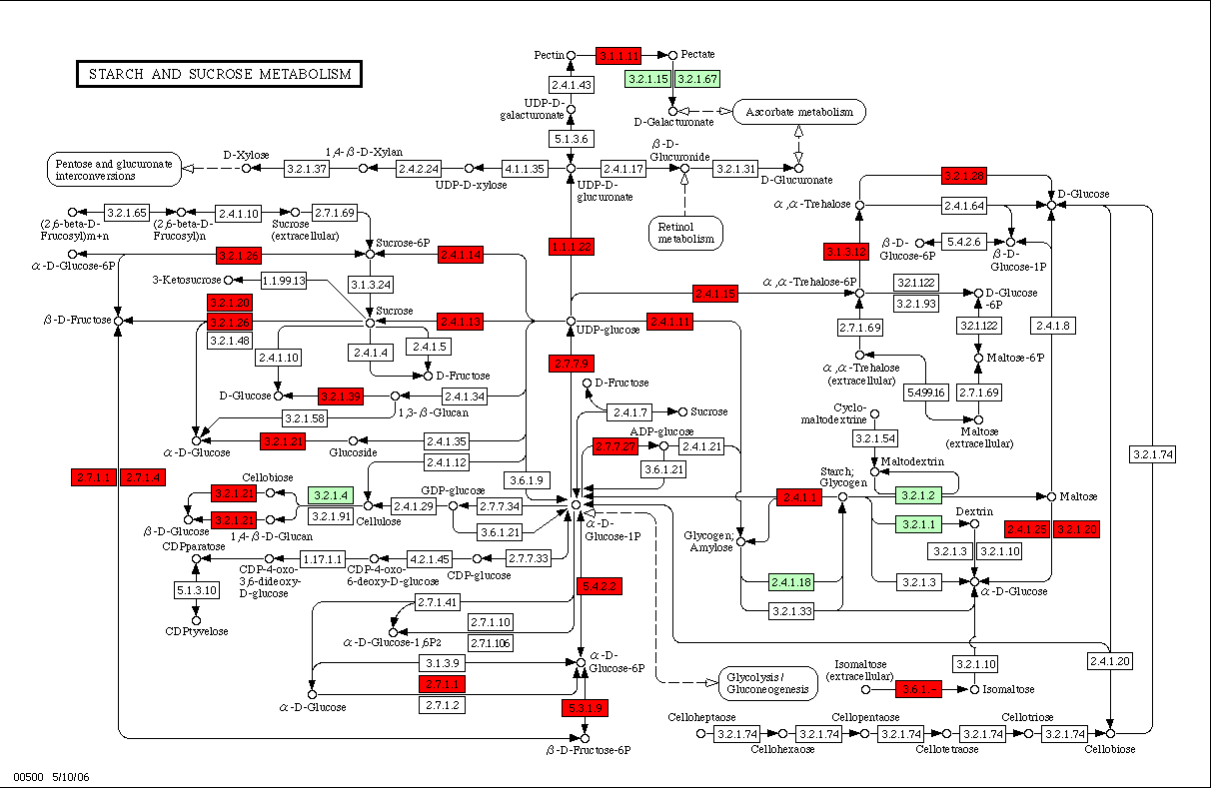
Pathway map of starch and sucrose metabolism. Sequences presumed to have been captured in the full-length library are shown in red. Arabidopsis genes not captured in cassava with this library are presented in green.

The 10577 different transcripts defined upon the assembly were annotated with gene function using the GoMp package (see "Methods"). Sequences were thus assigned Gene Ontology (GO) terms [40] and mapped to the Kyoto Encyclopedia of Genes and Genomes (KEGG) metabolic pathways [41] based on sequence similarity. Of the 10577 sequences, 8227 (78%) were annotated with terms of either of these controlled vocabularies, while 2350 (22%) had no function assigned. The use of the KEGG Orthology (KO) system [42] to annotate sequences allowed us to draw pathway maps of the transcripts in our library using Arabidopsis graphs as templates (Figure 2). We assigned cassava sequences to 101 of the 114 *A. thaliana *pathways, and according to the electronic annotation we may have captured about 60% (732 out of 1205) of the enzymatic activities (KO accessions) reported for Arabidopsis (Table 3).

**Table 3:** Comparison of the number of genes per pathway in Arabidopsis and in the full-length cDNA library according to the automated annotation. The 40 KEGG pathways with the largest number of cassava genes are presented.

**Accession**	**KEGG Pathway**	**Cassava Genes**	**Arabidopsis Genes**	**Pathway Coverage**
ath03010	Ribosome	89	110	0.81
ath00190	Oxidative phosphorylation	54	89	0.61
ath00195	Photosynthesis	41	69	0.59
ath00230	Purine metabolism	36	67	0.54
ath00240	Pyrimidine metabolism	29	54	0.54
ath03050	Proteasome	26	31	0.84
ath00010	Glycolysis/Gluconeogenesis	22	22	1.00
ath00710	Carbon fixation	22	24	0.92
ath00500	Starch and sucrose metabolism	22	29	0.76
ath00193	ATP synthesis	21	29	0.72
ath00620	Pyruvate metabolism	19	24	0.79
ath00970	Aminoacyl-tRNA synthetases	17	24	0.71
ath00251	Glutamate metabolism	16	23	0.70
ath00100	Biosynthesis of steroids	16	23	0.70
ath00400	Phenylalanine, tyrosine and tryptophan biosynthesis	15	24	0.63
ath00030	Pentose phosphate pathway	14	15	0.93
ath00020	Citrate cycle (TCA cycle)	14	15	0.93
ath00860	Porphyrin and chlorophyll metabolism	13	20	0.65
ath03020	RNA polymerase	13	21	0.62
ath00260	Glycine, serine and threonine metabolism	13	24	0.54
ath00252	Alanine and aspartate metabolism	11	16	0.69
ath00330	Arginine and proline metabolism	11	19	0.58
ath03022	Basal transcription factors	11	21	0.52
ath00670	One carbon pool by folate	10	11	0.91
ath00052	Galactose metabolism	10	12	0.83
ath03060	Protein export	10	13	0.77
ath00051	Fructose and mannose metabolism	10	14	0.71
ath00640	Propanoate metabolism	10	15	0.67
ath00350	Tyrosine metabolism	10	17	0.59
ath00071	Fatty acid metabolism	9	10	0.90
ath00630	Glyoxylate and dicarboxylate metabolism	9	12	0.75
ath00290	Valine, leucine and isoleucine biosynthesis	9	12	0.75
ath00510	N-Glycan biosynthesis	9	15	0.60
ath00380	Tryptophan metabolism	9	15	0.60
ath00910	Nitrogen metabolism	9	16	0.56
ath00900	Terpenoid biosynthesis	8	8	1.00
ath00941	Flavonoid biosynthesis	8	9	0.89
ath00360	Phenylalanine metabolism	8	10	0.80
ath00940	Stilbene, coumarine and lignin biosynthesis	8	11	0.73
ath00280	Valine, leucine and isoleucine degradation	8	16	0.50

For some pathways we captured the full-length transcript of genes homologous to more than 70% of the enzymes involved according to the Arabidopsis annotation, these almost-complete pathways include: 'Glycolysis/Gluconeogenesis' (100%), 'Starch and sucrose metabolism' (76%), 'Proteasome' (84%), 'Carbon fixation' (92%), 'Pyruvate metabolism' (79%), 'Biosynthesis of steroids' (70%), 'Pentose phosphate pathway' (93%) and 'Stilbene, coumarine and lignin biosynthesis' (73%) among others. The metabolic pathway of starch metabolism is of special interest in the case of cassava; the synthesis of this biopolymer is a relatively simple process that relies on the activities of three major enzymes: ADP glucose pyrophosphorylase (ADPGPase, 2.7.7.27), starch synthase (SS, 2.4.1.11) and starch branching enzyme (SBE, 2.4.1.18) [43]; as shown in Figure 2, we captured the full-length sequence of ADPGPase and SS, the pathway visualization also indicates that the SBE was not found in the library. Three cassava transcripts of ADPGPase were identified; these included one sequence of the small subunit of this enzyme and two alternative splicing variants of the large subunit. For the SS enzyme we found five sequences, these appear to be alternative transcripts of two enzyme isoforms.

Molecular markers are an important tool for crop improvement. Using the SSRFinder set of Perl scripts [44] and the AutoSNP package [45], we designed 1391 Simple Sequence Repeats (SSR) and 2356 Single Nucleotide Polymorphism (SNP) markers for 1725 of the 10577 captured transcripts; these markers were stored in a relational database where they were linked to the functional annotation of the sequences. After this process we got either a SNP or a SSR marker for 7 of the 22 cassava transcripts identified as enzymes in the starch and sucrose metabolism pathway, these enzymes include SS, starch phosphorylase (2.4.1.1), sucrose phosphate synthase (2.4.1.14) and UDP-glucose 6-dehydrogenase (1.1.1.22), which are enzymes known to have an effect on starch production [46,47]. Of the remaining 1718 genes associated with molecular markers, 563 were inside genes included in 85 different pathways.

To recognize stress inducible genes in this remarkably tolerant crop, we compared our sequences to the collection of drought and cold induced genes identified with the RIKEN Arabidopsis full length (RAFL) cDNA microarray [33]. Table 4 shows genes from that experiment with significant hits in our library; for 44 stress-induced genes in Arabidopsis, we captured 181 cassava transcripts showing significant sequence similarity (e-value < 1e-10) to 32 of them. Those genes for which we found more cassava transcripts include enzymes in the following categories: Aquaporins, endoxyloglucan transferases, beta-glucosidases, thiol proteases, heat shock proteins (HSPs), ascorbate peroxidases, thioredoxins, ethylene responsive element binding (EREB)/AP2-like proteins and catalases.

**Table 4:** Arabidopsis stress-induced genes identified by the RAFL microarray [33] captured in the cassava full-length library.

**Arabidopsis Gene**	**Accession**	**Description**	**Cassava transcripts**
FL5-3E18	ATHERD10	Aquaporin homolog	17
FL3-5J1	AB004872	Gamma tonoplast intrinsec protein 2	14
FL5-3P12	AB039929	EXGT-A2	13
FL5-2E17	AB039928	Beta-glucosidase homolog	12
*rd19A*	AB039927	Thiol protease	11
FL5-3J4	ATHRD19A	Heat shock protein dnaJ homolog	11
FL5-2123	AB050546	Ascorbate peroxidase	11
FL3-2C6	AB044404	Thioredoxin	10
DREB1A	AB050557	EREBP/AP2 protein	9
FL2-5G7	AB050558	Catalase 3 (CAT3)	8
FL2-1C1	AB050576	Cysteine proteinase homolog	8
*erd3*	AB050560	-	6
FL5-2122	AB050542	DC 1.2 homolog	6
FL5-1N11	AB050561	Non-specific lipid transfer protein	5
FL3-27	AB050562	Cysteine proteinase inhibitor homolog	5
FL5-95	AB050563	Rice glyoxalase 1 homolog	4
FL5-94	AB050550	Enolase	4
FL2-5A4	AB050564	DEAD box ATPase/RNA helicase protein (DHR1)	4
FL3-5A3	AB015098	Putative cold acclimation protein	3
FL5-2G21	AB044405	Reversibly glycosylated polypeptide-3	3
FL5-1A9	AB046991	Nodulin-like protein homolog	3
FL5-90	AB050565	β-amylase	3
FL3-3B1	AB050566	Hypothetical protein	2
*erd10*	AB050567	Group II LEA protein	1
*rd17*	AB050568	Group II LEA protein	1
*erd7*	AB050571	-	1
*erd4*	AB050551	Membrane protein	1
FL5-1F23	AB050573	Pyrroline-5-carboxylate synthetase	1
FL5-3M24	AB007787	LEA protein SAG21 homolog	1
FL5-1O3	AB050574	-	1
FL1-159	AB050575	HVA22 homolog	1
FL2-1H6	AB050552	Jasmonate-inducible protein homolog	1

### Gene correspondence and in-paralog (co-ortholog) detection

In the following sections the term ortholog will be used to designate sequences that are derived from a single ancestral gene in the last common ancestor of the species that are being compared [35]. This definition allows for cases were a single copy of a gene exists in each of these genomes (one-to-one orthologs) and cases where recent gene duplication has occurred and two or more genes in one species are orthologs with a single gene in another. In the later case, genes produced by gene duplication after a speciation event are called in-paralogs and they are co-orthologs of the corresponding gene in other species.

It is not our objective to provide a full classification of the transcripts captured in the full-length library into orthologs and paralogs, but to make use of the methods available to describe some interesting features of this collection of genes. First we use blast to designate pairs of genes that are reciprocal best hits (RBHs) when cassava transcripts are compared to those of other species. With this approach, RBHs are interpreted as potential one-to-one orthologs whereas co-orthologs are ignored; this way we are able to look for GO terms overrepresented in the set of sequences that remain unpaired (including possible gene duplications and alternative transcripts) as a means to recognize functional categories that are particularly frequent in the annotation of cassava transcripts. Second, we use blast to identify putative in-paralogs from a set of sequences from which alternative transcripts have been removed; this way we can produce a list of potential recent gene duplications for further analysis.

**Figure 3:**
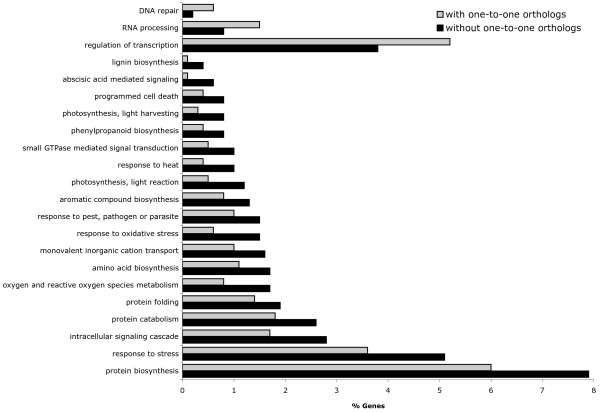
Comparison of the annotation of 6566 cassava sequences with putative one-to-one orthologs and 4313 sequences without. The Gene Ontology terms overrepresented and under represented (p-value < 0.05) for the sequences shared between cassava and *A. thaliana, P. trichocarpa *or *E. esula *are presented according to legend. GO terms related to stress response are frequent among cassava genes without one-to-one orthologs in any of these three species. 302 redundant sequences produced by CAP3 were included in the analysis.

The RBH criterion was used to define one-to-one orthologous pairs of genes between cassava and three other species: *R. communis*, *P. trichocarpa*, and *A. thaliana*. We found 3280, 5392 and 4678 shared sequences respectively. Then, to assess the function of the sequences that under these terms were found only in cassava, we compared the GO annotation of the sequences that were assigned to an orthologous pair and the annotation of those that were not. As a result (Figure 3), the GO terms enriched with cassava sequences (p-value < 0.05, Pearson Chi-square test) that were not assigned to a one-to-one pair included: 'protein biosynthesis', 'cellular protein catabolism', 'hormone mediated signaling', 'aminoacid biosynthesis', 'response to pest, pathogen or parasite' and 'lignin biosynthesis' among others. On the other hand, GO terms enriched with sequences assigned to an orthologous pair included: 'DNA repair', 'regulation of transcription' and 'RNA processing'.

Besides GO terms that are immediately associated to stress response like 'response to high light intensity,' 'response to heat' or 'response to oxidative stress,' sequences without a reciprocal best hit were frequently annotated with terms related to the synthesis of stress-responsive molecules like 'phenylpropanoid biosynthesis' [48]; also they were annotated with terms describing cellular processes that are enhanced during stress such as 'ubiquitin-dependent protein catabolism' [49,50] and 'abscisic acid mediated signaling' [51]; or, as a third example, with terms like 'photosynthesis, light harvesting', which we found to include mainly homologues of chlorophyll binding proteins, that might help protect the photosystems during high-light stress [52].

Given that many of the sequences without assigned orthologs were somehow involved in response to stress, we wanted to see if those unmatched sequences corresponded to recent gene duplications of stress-related genes instead of alternatively spliced forms or assembly errors of single genes. For this we excluded from our set of sequences the scaffolds that were identified as alternative splicing variants of other sequences. Then, we defined in-paralogs as sequences that were similar to each other and shared the same best hit in another genome (see "Methods").

**Figure 4:**
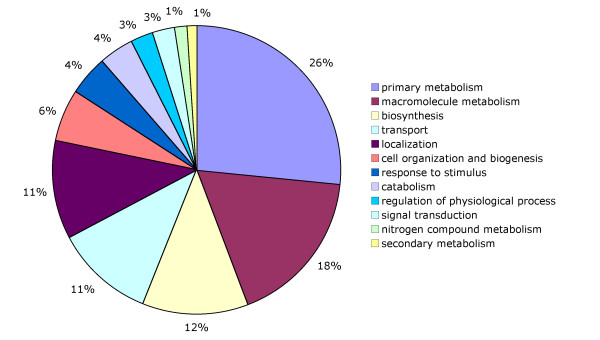
Main GO categories in the annotation of 230 potential gene duplications in cassava.

**Figure 5:**
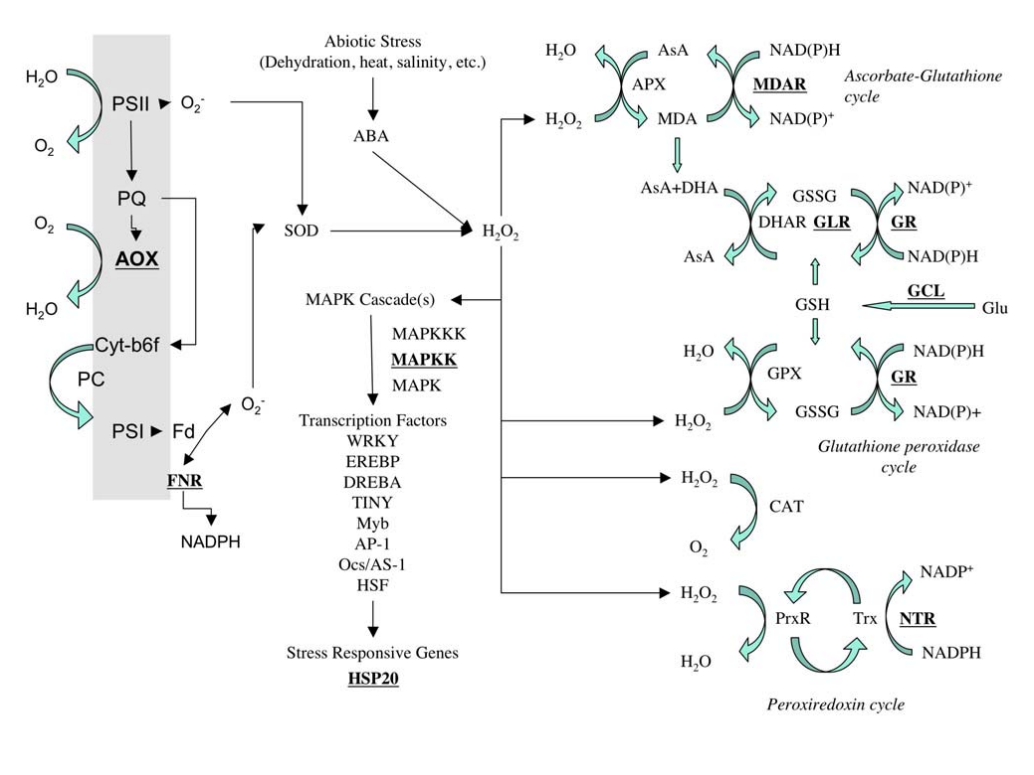
Reactive oxygen species processing in plant cells. Possible gene duplications in cassava are shown in bold and underlined. AOX, alternative oxidase; FNR, ferredoxin NADPH reductase; MAPKK, mitogen activated protein kinase kinase; MDAR, monodehydroascorbate reductase; GLR, glutaredoxin; GR, glutathione reductase; GCL, glutamate cysteine ligase; NTR, NADPH thioredoxin reductase; HSP20, heat shock protein 20; PSII, photosystem II; PQ, plastoquinone; Cytb6f, cytochrome b_6_f; PC, plastocyanin; PSI, photosystem I; Fd, ferredoxin; SOD, superoxide dismutase; ABA, abscisic acid; AsA, ascorbate; APX, ascorbate peroxidase; MDA, mohodehydroascorbate; DHA, dehydroascorbate; DHAR, DHA reductase; GSSG, oxidized glutathione; GSH, glutathione; Glu, glutamate; CAT, catalase; PrxR, peroxireductase; Trx, thioredoxin; Based on [72, 75, 104]

Using this approach and the additional restrictions mentioned in the methods section, we found 230 possible gene duplications; the GO annotation of these sequences is presented in Figure 4, most of them are homologous to enzymes involved in primary metabolism and macromolecule modification, however, there are several of these duplications in the 'response to stimulus' category. A closer look at this sequences revealed that enzymes such as monodehydroascorbate reductase (MDAR), glutaredoxin (GLR), glutathione reductase (GR), glutamate cysteine ligase (GCL), ferredoxin NADP^+ ^reductase (FNR) and NADPH thioredoxin reductase (NTR), seem to be duplicated; as shown in Figure 5, these enzymes catalyze important steps in reactive oxygen species (ROS) scavenging pathways, moreover enzymes like a mitogen activated protein kinase kinase (MAPKK) and heat shock protein (HSP20) that were also duplicated, are known to play important roles in stress response [53]. Multiple sequence alignments and the construction of parsimony trees for the sequenced regions of these genes support the idea of lineage specific expansions in cassava (Data not shown).

## Discussion

### Value of the cassava full-length cDNA library

We built the first EST characterized full-length cDNA library of cassava, providing nearly the same number of sequences previously available in EST databases of this species. The high number of novel sequences captured in this library can be taken as an indication of how poorly characterized the cassava transcriptome is; our library was not normalized, however, the fact that we extracted mRNA from leaves and roots of cassava plants under different environmental conditions, resulted in a low-redundancy set with more than 7000 distinct sequences represented by just one clone (Figure 1). This low redundancy could be the outcome of different gene expression patterns in response to the varying conditions used to build the library, also, the small overlap between our set of ESTs and those of previous efforts that focused on cassava traits like starch content and response to pathogens [25], could be an indication of the presence in our library of many genes specific to the abiotic stresses used in this study.

Full-length cDNAs are useful for the detailed annotation of sequence features in coding sequences and untranslated regions (UTRs) [30]. While the analysis of the first can sometimes render valuable information about protein structure and function through the annotation of amino acid motifs or protein domains [54], UTR sequences can be useful for the analysis of gene expression by means of the identification of transcription factor binding motifs [55], polyadenylation signals [56] and other structural features. Given the above, the importance of our effort is not only measured in terms of the amount of sequences captured, but also in terms of the quality and relevance of the genes represented in the library. We found that approximately 85% of the clones in our library contain full-length inserts; although this means that some of the cloned fragments are incomplete, the functional characterization of partial cDNAs in the library still allows the retrieval of sequence data for further experiment design and for the isolation of the full-length cDNA of specific genes. Moreover, from the EST information alone, we were able to determine the 5'UTRs of 1949 sequences and the 3'UTRs of 2241 sequences, as well as the complete coding sequence of 732 genes by running BLASTX against a set of known proteins, this information can be valuable to look for functional features such as micro RNA binding sites [57].

We tried to minimize annotation errors by using curated databases of protein function to retrieve GO and KO terms (see "Methods"). Although this can prevent the propagation of such errors, sequence similarity does not always guarantee functional relationship, especially when identity is low [58]. In our dataset, only 15 percent of the alignments that were used to retrieve functional annotations had a percent identity below 50 % and more than 70 % of the times the e-value was less than 10^-30^; as shown by Joshi and Xu [58], this level of sequence similarity can be expected to provide a 70 to 80 percent probability that two proteins will have similar functions, even for the most specific GO terms. Wilson and collaborators [59] have also showed that precise function is generally well conserved when sequence identity is above 40%. We trust that the overall representation of functional categories of the sequenced transcripts should not be very different from what we presented, however, at the more specific levels, one should be very careful in verifying the functional significance of sequence similarity.

Putative functions were assigned to 78% of the sequenced clones, this is in contrast with previous cassava EST collections for which as much as 63% of the sequences showed no significant similarity to known proteins [25], the high number of annotated sequences in our library may be due to an increase in the number of annotations in GO. Compared to similar reports in other species, we assigned a function to more sequences than those reported for maize [56] or wheat [29] full-length libraries, in these cases the amount of sequences with no function assigned were 52 and 44% respectively. The fact that a large portion of the sequences in our library has been assigned a function through sequence similarity aids the detection and isolation of particular genes known to participate in relevant biological processes, or at least of genes with features such as protein motifs that would make them interesting targets for research.

While most of the clones were linked to a molecular function or biological process using GO, the use of KEGG pathways to visualize functional assignments allows a much easier assessment of the enzymatic activities and metabolic processes for which we have transcripts. We mapped our cassava sequences to almost all of the pathway graphs of Arabidopsis; it is noteworthy that with only 10577 distinct transcripts, the equivalent to more than half of the pathway knowledge represented in KEGG for Arabidopsis been inferred from electronic annotation in cassava. KEGG pathways consist of reference diagrams on top of which species-specific enzymes can be drawn, since not all the metabolic pathways are as conserved as to allow the construction of a reference diagram, most of the KEGG pathway graphs are of intermediary metabolism processes, and only a few regulatory pathways for a particular species like *A. thaliana *are available [42]. Nonetheless, traits of agronomical value such as starch content and quality [6], carotene production [60], photosynthesis [10] and lignin biosynthesis [61] that are important targets for cassava improvement are easily related to some of the KEGG pathway maps (Figure 2); identification of cassava genes participating in these as well as other processes would allow a rapid selection, isolation and characterization of key enzymes for the improvement of the crop, i.e. rate limiting enzymes or catalytic elements missing from a biosynthetic pathway. As an example, ADPGPase has been shown to be a rate limiting enzyme in starch biosynthesis whose over-expression in cassava leads to increased root biomass [62], the characterization of the molecular diversity of this as well as other enzymes could lead to the development of higher yielding crops.

Although our library was oriented towards stress genes, the fact that we captured the full-length transcript of important enzymes in processes such as starch biosynthesis shows that we have a valuable resource for several aspects of cassava research. Furthermore, since we were able to design molecular markers for genes that directly or indirectly affect the poising between starch production and the synthesis of other molecules like sucrose [47] or glucuronate; it is possible that the information provided by this library will provide elements for marker assisted selection in this as well as other processes, i.e. protein biosynthesis, carotene accumulation, disease resistance, etc.

Once a candidate gene in cassava is selected, the complete cDNA sequence can be easily isolated from the corresponding clones. This sequence can then be used to screen the molecular diversity of the studied loci to find gene variants suited for molecular marker development in a breeding program; with this in mind, we used the ESTs to design SSR and SNP molecular markers; if these markers are within interesting genes, then they could be valuable for the detection of quantitative trait loci (QTL). Moreover, once the cassava genome is revealed, they could serve as a tool for pseudomolecule assembly [63], which is an addition to the central role of the full-length sequences in gene annotation.

We found that our library contains transcripts of 7796 different genes, this is a small number compared to the expected number of genes for a higher plant [63,64]; The calculated number of 50698 transcripts in cassava is a guide for the future efforts required for the completion of the gene catalog for this species. This figure however may well be an overestimation of the true number of transcripts, mainly because our library is believed to be rich in rare transcripts that result from specific stress conditions [39].

### Transcripts of stress-related genes

RNA from different tissues of plants under normal conditions was pooled with that of plants under PPD, drought, heat and acidic soil stresses, we therefore suppose that our library should be enriched with transcripts of genes induced by these conditions. If this is the case, one of the objectives of the analysis should be the identification of the types of stress-induced genes in the library. As stated before, the abundance of novel cassava sequences in our set of transcripts could be an indication of the specificity of the genes captured according to the different treatments; this assumption is supported by the fact that we captured most of the stress-induced genes detected by the RAFL microarray [33] and that most of the sequences without assigned one-to-one orthologs in other species are in GO categories linked directly or indirectly to stress response.

The comparison of the annotation of sequences with and without RBHs that we made for the detection of stress-induced genes is based on the following assumption: if there is more than a spliced form, allele or copy of a certain gene in a set of sequences, the use of the RBH criterion to define orthologs in a second set would assign just one of them to the corresponding sequence in the second group, this would leave an amount of unpaired sequences that would enrich the corresponding GO category in the group of sequences without orthologs. If our library includes many transcripts of stress related genes, then it is more likely to find alternative splicing variants or even more assembly errors of these sequences; this would lead to an overrepresentation of genes annotated with GO terms corresponding to the functional categories of these sequences.

One possible explanation for the fact that many of the sequences without one-to-one orthologs were associated with stress-related genes could be the existence of recent gene duplications of these sequences in the cassava lineage. We applied a very conservative methodology to detect some of these duplications (see "Methods"); with this method, two or more cassava sequences sharing their best hit in at least two other genomes are considered as potential in-paralogs. In order to create a method to establish gene correspondences across genomes while dealing with recent and ancient gene duplications, Kellis and collaborators have defined a best unambiguous subset as a group of genes such that all best hits of any gene within the set are contained within the set and no best hit of a gene outside the set is contained within the set [65]; accordingly, we defined potential gene duplications in cassava by finding pairs of potential in-paralogs in which only one of the sequences was a best hit of genes in a second genome; in this way we avoid the report of false positives in which orthologs exists for the candidate in-paralogs, but both best blast hits point to just one of them [66], in many cases as a result of different sequence lengths.

As shown in Figure 4, almost 5% of these potential duplications are annotated in the 'response to stimulus' GO category; although this number may seem small, we have already seen that not all of the sequences related to stress response are annotated in that category. For instance, many sequences involved in ubiquitin-mediated proteolysis correspond to a large portion of the duplications in the 'macromolecule metabolism' category. A detailed inspection of the 230 possible gene duplications that we found, revealed that they were homologous to several enzymes related to ROS metabolism; since heat [67], drought [68], acidic soils [69] and PPD [70] have been reported to induce ROS production, we think that these potential duplications are a good example of how the full-length library can provide hints as to the mechanisms underlying cassava stress response features.

Most ROS in plants are produced by dismutation of superoxide generated by electron transfer to molecular oxygen in the Mehler reactions of the chloroplast [71]. During stress response, H_2_O_2 _production can be increased [72] in a process that sometimes involves abscisic acid mediated stomatal closure and reduction of CO_2 _levels for photosynthesis [73]. High dosage of hydrogen peroxide results in hypersensitive cell-death while low quantities of this molecule can trigger a protective function against different stress conditions [74], this ROS-mediated activation of stress responses is dependent on a network of many genes that balance the outcome of ROS-scavenging and ROS-producing proteins [75]. What we found in our sample of the cassava transcriptome is that many important enzymes in the main pathways of H_2_O_2 _scavenging seem to be duplicated (Figure 5); we propose that these duplications may account, at least in part, for the stress tolerance characteristics of cassava, and that this could be due to a tighter control of some of the ROS signaling mechanisms already described for other plants. We base our hypothesis on the extensive literature regarding the importance of molecules like MDAR [76], FNR [77], NTR [78], GLR [79,80], thioredoxin [81] and glutathione [82,83] in plant sensitivity to environmental stress.

It is believed that the genus Manihot emerged by recent allopolyploidization, this event is considered responsible for both, rapid speciation and weak interespecific barriers leading to hybridization [84]. We hypothesize that potential duplications of stress-responsive genes could have originated in this polyploidization event; since it has been shown that most genes are rapidly silenced after these episodes unless they diversify in function [85], it would be interesting to see if some of the detected duplications show evidence of subfunctionalization. A first step to do this would be the evaluation of the expression profiles in different organs and under various conditions of the plausible gene duplications detected with this library, this could readily be done through microarray construction, for which once again the full-length library would be an invaluable resource [33].

## Conclusion

The cassava research community will certainly benefit from the full-length library here presented. The analysis of the sequenced clones already suggests tempting research directions for the improvement of this crop. An in-depth analysis of gene features and gene families in cassava, as well as the fine-tuning of their assigned functions, could provide the necessary elements for the enhancement of production under stress environments, moreover, what we learn from this crop should lead to important achievements in other important but not so tolerant plant species.

## Methods

### Plant material and abiotic stresses

Total RNA was extracted following a protocol based on the method reported by Chang *et al.*[86] from cultivar MTAI16 of cassava plants under the conditions depicted in Table 5. The plants used for RNA extraction at different time points and for abiotic stress treatments were grown in plastic pots in a green house; nine month old plants were harvested directly from the field.

**Table 5:** Conditions and tissues used for mRNA extraction.

**Treatment**	**Age**	**Tissue**	**Duration of treatment before RNA extraction**
No treatment	9, 11, 12 weeks	leaf	
No treatment	9 month	root	
Drought shock	7-weeks	leaf	3, 6, 24, 72 hours
Heat	9-weeks	leaf	3, 6, 24, 72 hours
PPD	9 month	root	24, 48, 120 hours
High Al, low pH	9 weeks	leaf	3, 6, 24, 72 hours
High Al, low pH	9 month	root	6, 24, 48 hours

For the high Al-low pH treatment, plants were placed in continuously aerated solutions containing 200 μM AlCl_3 _and 200 μM CaCl_2 _with pH 4.2. In the heat treatment they were incubated at a temperature of 42°C. For drought shock, plants were taken out of the pots and then their roots were washed with water, dried with a towel and left at room temperature. For the PPD treatment, nine-month-old roots were harvested and their distal and proximal parts cut, the proximal part of the tuber was covered with plastic, and the root was cut in 2 cm slides before RNA extraction.

### RNA preparation and construction of the full-length cDNA library

Poly (A)^+ ^RNA was prepared with the μMACS mRNA Isolation Kit (Miltenyi Biotec) under standard conditions given in the manual. A full-length cDNA library was constructed from the poly (A)^+ ^RNA by the biotinylated CAP trapper method using trehalose-thermoactivated reverse transcriptase [31]. The resultant double-stranded cDNAs were digested with *Bam*HI and *Xho*I, and ligated into the *Bam*HI and *Sal*I sites of a Lambda FLC-III vector [87].

### EST sequencing

The DNA of each clone was directly amplified from 384 bacterial cultures of a glycerol stock plate by the RCA method [88] using a TempliPhi HT DNA amplification kit (GE Healthcare, United Kingdom). End sequencing of 19968 clones was carried out using ABI 3700 automated capillary DNA sequencers (Applied Biosystems). The M13-21 primer (5'-TGTAAAACGACGGCCAGT-3') and the 1233 primer (5'-AGCGGATAACAATTTCACACAGGA-3') were used for forward and reverse sequencing, respectively.

### Trimming of sequence data and assembly

Raw sequence data was base-called using the Phred program [89], the low quality region (Phred quality score < 20, and more than 20 bases repeated) which was found at edges of each raw sequence was discarded. For vector sequence detection, we used the sim4 program [90]. Sequence data of a length shorter than 100 bases after this trim process was omitted. In addition, if the repetition of a single nucleotide in a sequence was longer than 10% of its total length, we rejected such a sequence. ESTs were assembled by CAP3 [91] with default parameters. Trimmed sequences were submitted to DDBJ under accession numbers DB920056-DB955455 while trace files were uploaded to the trace archive [92] under accessions 1918207201 to 191824260.

### Full-length cDNA library quality

We calculated a full-length ratio with sample clones satisfying the following conditions: A clone had both, 5' and 3' sequence data, the e-value in a 5' sequence fastx34 search [93] was less than 1e-30 against the NCBI-nr dataset, and the aligned frame was in a plus direction. The clones fulfilling the conditions mentioned above were identified as 'full-length' if the fastx34 alignment of 5' sequence data started with methionine and a poly (A)^+ ^tail existed in the 3' sequence.

### Scaffold construction

In order to obtain a non-redundant set of transcripts, these were clustered according to clone names. For this, the '.ace' file from the CAP3 output was parsed to build scaffolds, that is, groups of sequences representing a unique transcript for which the relative position and orientation of the fragments can be inferred. Using clone names, the contigs or singletons corresponding to the two ends of a given clone were joined together by adding 20 Ns in the middle of both sequences. Since 20 is the default window size in BLAST searches these Ns do not interfere with the BLAST analyses.

### Functional annotation of the sequences

Once these scaffolds were created, the sequences were introduced to a pipeline previously developed at CIAT for functional annotation of DNA sequences (GoMp). Shortly, the system uses protein sequences in high quality curated annotation databases, GO lite [94] and TAIR [95], and transfers the respective GO and KO annotations to sequences showing significant sequence similarity (e-value < 1e-5) using BLASTX [96]. Within the top five hits of the blast report, we look for the best alignment with a sequence having assigned GO terms and a sequence with assigned KO accessions, if these two sequences are not the same, then both annotations are reported in order to assign a functional annotation from both controlled vocabularies. Annotations are then stored in a relational database and become navigable through web cgi-scripts. Pathway maps were drawn based on the KO accessions using the KEGG web services API [96].

### Identification of alternative transcripts

To identify alternative splicing variants we used two approaches. First, we used the resulting CAP3 assembly and clone id information to select groups of two or more contigs formed by sequence reads of clones with a second read assembled in only one contig. Secondly, we performed a BLASTN analysis of the remaining sequences against itself and filtered those pairs for which all high scoring pairs of more than 100 base pairs had a percent identity of more than 98%.

### Annotation of coding sequences and UTRs

For the annotation of coding sequences and UTRs, the blast report that was used to assign functions to the sequences was parsed to extract alignments around the beginning or end of known proteins, these positions were then used to find, in the same reading frame, start or stop codons that would define the start and end points of coding sequences and UTRs in the ESTs.

### Molecular marker design

We used the SSRFinder set of Perl scripts [44] in order to find microsatellite repeats and automatically design PCR primers around them. Additionally, the e-PCR software [97] was used to remove unspecific primer pairs that annealed to more than one transcript.

The AutoSNP software [45] was used to find single nucleotide polymorphisms. For this we created a second CAP3 assembly including our sequences and ESTs from cassava accessions MBRA685, MPER183, CM523-7, MCOL1522 and SG107-35. We designed primer pairs around these SNPs using primer3 [98] and primers for single base extension using the SBEprimer package [99].

### Gene prediction in *R. communis*

The published assembly of the *Ricinus communis *genome (GenBank accessions AASG01000001 to AASG01036140) was masked for repetitive elements using RepeatMasker [100] and *Arabidopsis *repeat libraries. All Euphorbiaceae ESTs in GenBank (October 2006) were used to train TIGRSCAN for gene prediction [101] on the masked sequences. Putative protein coding genes having ORFs larger than 200 base pairs were considered; this resulted in a set of 22734 predicted genes used in further comparative analysis with *M. esculenta*. Although being a draft shotgun genome (129 MB out of ~300 MB, 2x coverage, maximum contig length 36140 and N50 length = 3683), it was still deemed valuable for analysis as it is currently the most complete genome sequence belonging to the Euphorbiaceae.

### Comparative genomics analysis

The sequences of the predicted and/or verified transcripts of *Arabidopsis thaliana*, *Populus trichocarpa *and *Ricinus communis *were downloaded or determined from the genomic sequences. Then several reciprocal blast analyses were run using TBLASTX, e-value < 1e-5, and parsed to retrieve the best hit of each sequence of cassava in each of the other databases and the best hit of each sequence of the other species in cassava. We thus built a table representing the network of best hits among the four species.

A one-to-one ortholog was identified as a reciprocal best blast hit [102], with this definition we were able to divide our set of sequences into those that had one-to-one orthologs in any other species and those that did not. The comparison of the annotation of the two sets of sequences was performed using the WEGO web server [103], terms overrepresented in one of the two groups (p-value < 0.05 Pearson Chi-Square test and more than 5 genes per category) were considered for further analysis as having an overabundance of transcripts.

In-paralogs were found based on the same network of blast results with the following definition: C1 and C2 are cassava sequences, C1 and C2 are BLASTN hits (e-value < 1e-10) of each other and the best hit of both sequences in at least two of the datasets used for the comparison corresponds to a single gene. If the best hit of C1 and C2 in dataset A is sequence A1 and their best hit in dataset B is sequence B1, then the best hit of A1 and B1 in cassava must be either C1 or C2. Finally, for all C1 and C2 pairs fulfilling the above conditions, if only one of them is a best hit of sequences in dataset A and only one of them is a best hit of sequences in dataset B, then they are considered in-paralogs.

## Authors' contributions

TS Conducted bioinformatics analyses and drafted the manuscript, GP and FR were responsible for the conception and execution of the computational analyses, the interpretation of the results and co-wrote the manuscript, AS carried out the abiotic stress treatments and prepared the RNA for library construction, AI provided bioinformatics assistance for the processing of sequence data, AT and YS conducted sequencing of the cDNA clones, MS and KS participated in the coordination of the analysis and helped to draft the manuscript, JT and MI conceived the study and critically revised the manuscript. All authors read and approved the final manuscript.
